# Some of the Difficulties in the Diagnosis of Sarcoma of Bone

**Published:** 1892-03-26

**Authors:** 


### SURGERY.

### SOME OF THE DIFFICULTIES IN THE DIAGNOSIS OF SARCOMA OF BONE.

It is often a very difficult, and, of course, always a most important matter to distinguish between sarcoma of bone and an inflammatory swelling or necrosis. Sometimes it is impossible to do so with the most careful diagnosis. This difficulty is well illustrated by the following case which we have under observation in hospital.

A boy ten years of age came in a month ago, with a history of pain and swelling about the lower part of the thigh of two weeks' duration. He was said, as so commonly happens, to have had rheumatic fever, but this pain and swelling of the lower part of his thigh were the only symptoms. The pain had not been severe. There was no history of injury. On admission, the lower half of the femur was enlarged all round, there was very little tenderness, and no redness of the skin; and the boy did not seem in pain. There was no fluctuating area. The knee joint, though slightly limited in its range of movement was normal. The thickening of the bone was uniform and not very marked. During the month he has been in the hospital, *e.g.*, up to the present time, the enlargement of the bone has increased, and two distinct hard bosses have formed, one on the anterior, the other on the inner aspect. The range of movement in the knee became more limited, but was quite free within a given range, and there was no sign of fluid in the joint. There was a rise of temperature to 100 deg. every night. The boy did not suffer pain in the swollen bone, and it was only very slightly tender. The condition of the limb assumed a very suspicious form, and notwithstanding the temperature, one could not help suspecting a sarcoma expanding the lower end of the bone. We have seen a persistent temperature of 100 deg. at night in a rapidly growing periosteal sarcoma of the femur. Presently the skin on the outer side of the femur in the lower third got dusky red and brawny, and a few days later fluctuation could be detected there. An incision was made, and a semi-solid mass of lymph the size of a walnut evacuated. On passing the finger through the wound, a small sequestrum, about the size of a shilling, was felt firmly fixed in the under surface of the lower end of the femur. Until suppuration occurred, and the finger was guided by the suppurating track to the seat of necrosis, there was no guide for exploration. Indeed, an exploratory incision had been made on the anterior aspect at an earlier period in the case, and nothing found.

It is remarkable how little pain there is in some of these cases of quiet necrosis, a point of strong resemblance to malignant growth, and there is no redness of skin, and no signs of suppuration; indeed, the symptoms very closely resemble those of sarcoma. We must wait and watch these cases. In this one, after nearly two months, the occurrence of suppuration enabled us to make a correct diagnosis, after much suspicion of more serious disease. An exploratory incision will not always clear up the doubt. In Mr. Morrant Baker's classical case it rather obscured the diagnosis. A young man had a swelling on the femur which was thought to be either a sarcoma or one of these cases of quiet necrosis, and to try and settle the question an exploratory incision was made. But, unfortunately, the granulation tissue could not be distinguished from sarcoma and the limb was amputated for what was really only necrosis. Mr. Erichsen records a case diagnosed as scirrhus of the male breast, which turned out to be one of quiet necrosis of the rib, and here the thickened tissue was actually cut into and removed under the impression it was a scirrhus before the necrosis was discovered underneath.

Not only do cases of quiet necrosis very closely resemble malignant disease of bone, but cases of periosteal sarcoma sometimes resemble in many respects inflammatory affections of bone. Redness of the skin over the growth, effusion into neighbouring joint, and rise of temperature have all been observed in cases of periosteal sarcoma.

The diagnosis of chronic inflammatory thickening of the bone from sarcoma is often a matter of great difficulty. As a rule in chronic inflammatory thickening the pain is greater, pain especially at night. The egg-shell crackling of endosteal sarcoma would not be present in the early stage to help us. In all cases of doubt we ought to try iodide, in case we have an unrecognised syphilitic bone lesion to deal with.

A periosteal sarcoma often presents semi fluctuating bosses which have been mistaken for abscesses. We have lately seen a case in which a swelling with such bosses was diagnosed as necrosis, and the shaft of the radius torn out under the impression it was a sequestrum, lying in a mass of granulation tissue. The sarcoma continued to develop from the periosteum, and formed a large mass with fungating granular bosses and semi-fluctuating nodules, and the limb was amputated.

A periosteal sarcoma situated deeply in a limb, soft and semi-fluctuating, may be mistaken for an abscess, but the edge is more defined. In any case of suspected periosteal sarcoma of a soft semi-fluctuating character, we should always explore for pus with an exploring syringe.

We may be able to diagnose a sarcoma in some cases, but be very doubtful whether or not it is connected with the bone. A few years ago we watched such a case. One could move the growth on the bone quite distinctly, and yet the shaft seemed a little thickened in its neighbourhood. After removal we found the tumour growing from the outside of the periosteum, which was separated from the bone beneath by much soft friable sarcoma tissue, so that the tumour and the periosteum were not firmly fixed to the bone; the bone was much eroded by the sarcoma tissue beneath the periosteum.

There is a rare form of periosteal tumour which might be mistaken for a sarcoma, and that is periosteal lipoma, a fatty tumour growing from the outer layer of periosteum. A few years ago we had such a case under observation. The patient was a child aged two years, and came into hospital with a painless, gradually increasing swelling at the back of the thigh of four months' duration. On examination we found an undefined, soft, semi-fluctuating, moveable, swelling deep in the thigh just above the popliteal space. On cutting down on it, it was found to be the size of a hen's egg and to consist of fatty tissue growing from the periosteum. It was fairly encapsuled. Microscopically it was a typical lipoma. Chemically we could not make out its attachment to this bone, and therefore the question of diagnosis from periosteal sarcoma did not arise, but it certainly might in another case. Mr. D'arcy Power recorded a similar case at the Pathological Society in 1888, in a boy nine years of age. The tumour was growing from the periosteum of the upper part of the femur.

The possibility of a deeply-seated painful growth—apparently periosteal sarcoma—being a coagulated, non-pulsating, cured aneurism, pressing on a nerve, must not be forgotten.

The diagnosis of pulsating sarcoma of bone from aneurism, in situations where a large artery exists, is sometimes one of extreme difficulty. In both cases the pulsation is expansile, and in both there may be a bruit, and the possibility of diminishing the size of the swelling, with a rapid return of size and pulsation on removal of the pressure. If a pulsating swelling like an aneurism seems closely connected with the bone, the possibility of its being a pulsating sarcoma should be remembered.

